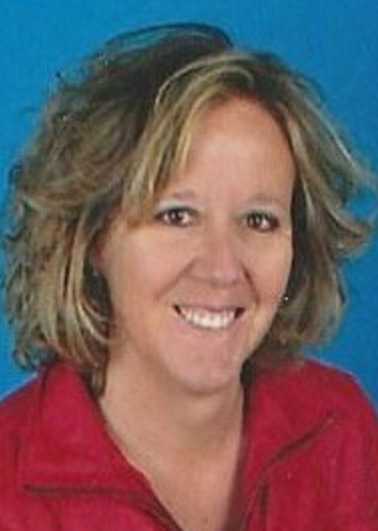# Self‐Assembled Porphyrinoids: One‐Component Nanostructured Photomedicines

**DOI:** 10.1002/cmdc.202100201

**Published:** 2021-05-19

**Authors:** Irene Paramio, Tomás Torres, Gema de la Torre

**Affiliations:** ^1^ Department of Organic Chemistry Universidad Autónoma de Madrid C/Francisco Tomás y Valiente 7 28049 Madrid Spain; ^2^ Institute for Advanced Research in Chemical Sciences (IAdChem) Universidad Autónoma de Madrid C/Francisco Tomás y Valiente 7 28049 Madrid Spain; ^3^ Instituto Madrileño de Estudios Avanzados (IMDEA)-Nanociencia C/Faraday 9 28049 Madrid Spain

**Keywords:** porphyrinoids, self-assembly, nanostructure, phototherapy, nanotheranostics

## Abstract

Photodynamic therapy (PDT) is becoming a promising way to treat various kinds of cancers, with few side effects. Porphyrinoids are the most relevant photosensitizers (PS) in PDT, because they present high extinction coefficients, biocompatibility, and excellent photochemical behavior. To maximize therapeutic effects, polymer‐PS conjugates, and PS‐loaded nanoparticles have been developed, with insights in improving tumor delivery. However, some drawbacks such as non‐biodegradability, multistep fabrication, and low reagent loadings limit their clinical application. A novel strategy, noted by some authors as the “one‐for‐all” approach, is emerging to circumvent the use of additional delivery agents. This approach relies on the self‐assembly of amphiphilic PS to fabricate nanostructures with improved transport properties. In this review we focus on different rational designs of porphyrinoid PS to achieve some of the following attributes in nanoassembly: i) selective uptake, through the incorporation of recognizable biological vectors; ii) responsiveness to stimuli; iii) combination of imaging and therapeutic functions; and iv) multimodal therapy, including photothermal or chemotherapy abilities.

## Graphical abstract

**PDT improved**: Self‐assembled photosensitizers (PS) built through the “one‐for‐all” approach can help to overcome the disadvantages of traditional nanodelivery systems. This minireview aims to show the potential of this emerging strategy highlighting different rational designs of porphyrinoid PS to achieve nanosystems with improved therapeutic effects, such as selective uptake, responsiveness to stimuli, nanotheranostics abilities and multimodal therapy.

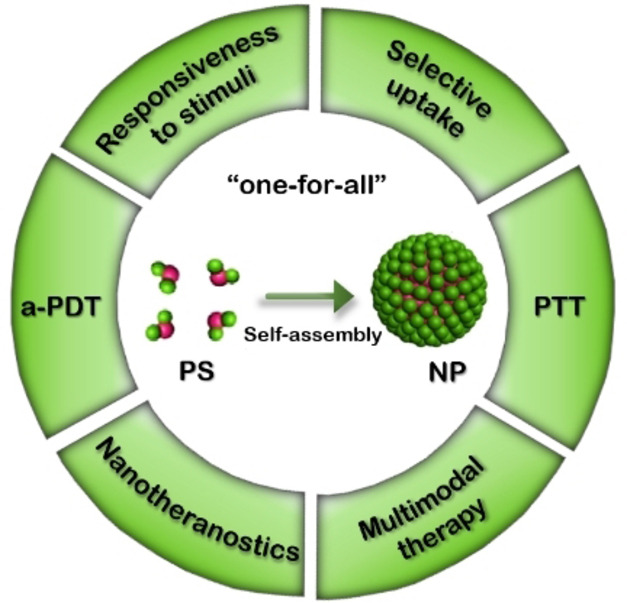

## 1 Introduction

Photodynamic therapy (PDT) is a non‐invasive form of phototherapy that utilizes harmless light to activate non‐ or minimally toxic photosensitive chemicals called photosensitizers (PS) to generate cytotoxic species for malignant cell eradication.[^1^, ^2^, ^3^, ^4^] Upon irradiation of light with appropriate wavelength, PS is activated to the nanosecond‐lived excited singlet state, which quickly converts to a more stable excited triplet state *via* intersystem crossing. The triplet PS exists long enough to generate cytotoxic reactive oxygen species (ROS) such as superoxide and hydroxyl radical (Type I mechanism), or to generate highly reactive singlet oxygen (^1^O_2_) through energy transfer to ground‐state triplet oxygen (^3^O_2_) (Type II mechanism), which causes damage to the tumor cells and vasculatures by apoptosis, necrosis, or activating the immune responses. PDT has received tremendous attention in the past decades due to its broad applicability (both to localized tumors and infections), high selectivity and spatio‐temporal resolution (only works under local illumination to the lesions). Moreover, its mode of action does not usually cause the emergence of resistance.

Over the past century, a large number of molecular PS were synthesized and investigated, aimed at overcoming issues such as poor solubility in aqueous media, short excitation wavelength (which derives in light attenuation due to absorption or scattering by the tissues), limited structural stability and non‐negligible dark toxicity.^[5]^ Porphyrinoid‐based derivatives are the most frequently used PS in PDT applications, because of their singular photochemical and structural characteristics.[^6^, ^7^, ^8^] A number of synthetic porphyrinoids (commonly called second‐generation PS), which include synthetic porphyrins (Por), chlorins (Chl) and phthalocyanines (Pc), have been developed over the last years, and some of them have been already approved for the treatment of different diseases. Overall, these chromophores feature: i) intense absorption inside the range of the optical therapeutic window (optical range with minimum absorption and scattering and better penetration depth, ranging from 650 nm to 850 nm); ii) high triplet quantum yield (Φ_T_) with relatively long lifetime (τ_T_), which assists in the generation of ^1^O_2_ in high quantum yield (Φ_Δ_); and iii) lack of dark toxicity in most of the cases.

Despite the progresses in PS development, PDT is still not implemented as a first‐line treatment of cancer because of non‐optimal outcomes related to the low uptake of the PS by malignant cells, lack of selectivity towards malignant tissues, and local hypoxia at tumor sites that arises from the disordered tumor growth. However, the use of nanostructured materials can help to overcome those critical obstacles. Prompted by the current explosion of nanotechnology, the development of nanomedicines in general, and “photonanomedicines” in particular, with insights into transport processes and biological responses, has grown exponentially.[^9^, ^10^, ^11^] Numerous nanostructured PS delivery systems, such as polymer‐PS conjugates and PS‐loaded nanoparticles, have been developed to enhance the therapeutic effects by improving bioavailability and tumor targeting efficacy.^[12]^ These drug delivery systems exploit either passive targeting strategies (enhanced permeation and retention, EPR),^[13]^ which allows nanomaterials to accumulate in tumors via leaky blood vessels, or active targeting that relies on the use of biologically recognizable vectors (antibodies, peptides, proteins, carbohydrates…) capable of interacting with specific receptors overexpressed on target cells.^[14]^ Another relevant advance in the development of more effective and specific treatments for cancer therapy is the application of nanotheranostic agents that possess simultaneous monitoring and therapeutic competencies.^[15]^

As mentioned above, the development of targeted photoactive nanomedicines has been mostly based to date on the derivatization of inorganic and polymeric nanoparticles conjugated with different elements that afford therapeutic/imaging effects, bioavailability, and tumor targeting delivery. This approach is referred as “all‐in‐one” because multifunctionality and theranostics are achieved by combining multiple components that each possesses a specific singular function.^[16]^ However, some problems derive from these “all‐in‐one” nanosystems. Inorganic materials suffer from non‐biodegradable features, while nanodrugs based on polymer nanoparticles are still subject to a lot of limitations concerning their multistep fabrication and low reagent loadings, limiting their further clinical applications. Moreover, these nanosystems require complex toxicity investigations due to the presence of multiple components and sometimes, the concentration of released PS at tumor sites is still low, causing an unsatisfactory PDT effect.

Alternatively, building biocompatible multifunctional nanosystems created by self‐assembly of photophysically active molecules may overcome the mentioned issues,^[17]^ since the photodrug acts as both the carrier and the cargo.^[18]^ This innovative approach noted by some authors as “one‐for‐all” design relies on the self‐assembly (driven by π‐π interactions, hydrogen bonding, hydrophobic forces, etc. ) of tailored PS, as a bottom‐up approach to fabricate nanostructures, circumventing the need of additional delivery agents. En route to fabricate spontaneously self‐assembled nanoparticles in aqueous media, amphiphilicity of the PS is an important structural feature to rely on.^[19]^ The combination of the natural organization of amphiphiles with the advanced concept of supramolecular self‐assembly,^[20]^ leads to the development of a wide variety of structures including micelles, vesicles, nanotubes, nanofibers among others.^[21]^ These nanostructures, with improved transport properties and selective uptake by target cells with regard to non‐assembled individual molecules are ideal for light‐controlled biomedical applications; indeed, the building blocks can be rationally designed for combining imaging and therapeutic functions, and also incorporate recognizable residues for active targeting. As aggregation of the π‐conjugated PS usually induces self‐quenching behavior and cancelled ^1^O_2_ generation abilities (except for *J*‐aggregates that can be both fluorescent and triplet state photoactive[^22^, ^23^]) it is important to remark that the PS must form stable nanostructures in the more hydrophilic extracellular media but disassemble at the cell membrane or at the membranes of the corresponding organelles where hydrophobia predominates to recover its photophysical activity.

The purpose of this review is to focus on the most important work that has been carried out in the last years with “one‐for‐all” self‐assembled porphyrinoid PS, encompassing aspects such as active targeting, activatable systems, nanotheranostics and combined therapies, the latter including the encapsulation of chemotherapy drugs inside the one‐component PS assemblies. Overall, this article aims at highlighting the benefits of this approach in terms of simplicity and therapeutic effects. Although MOFs built with PS ligands could be also considered as self‐assembled porphyrinoids,^[24]^ we will focus this review on the assembly of purely organic amphiphilic PS mainly driven by a combination of hydrophobic, van der Waals, and π‐π interactions.

## 2 Passive and Active Targeting

EPR effect is often used to describe passive delivery of anticancer drugs to tumors. In tumor pathology, new blood vessel formation results in abnormally constructed vessels with large vascular fenestrae, as large as 600 nm. As a result, particles less than 200 nm preferentially accumulate in the tumor interstitium.^[25]^ Nanoparticle‐based drug delivery systems with a defined size range of 10–100 nm typically demonstrate the most effective tumor penetration and reduced systemic toxicity compared to free drug formulations. In particular, self‐assembled nanoparticles combine the favorable biodistribution and pharmacokinetic properties of nanodelivery vehicles, and the rapid diffusion and penetration properties of smaller drug cargos.

Several porphyrinoid‐based macromolecules and/or nanoaggregates have exploited the EPR effect for the selective delivery of the PS to the tumor. For instance, a Por‐cored hyperbranched polymer that aggregates into large micellar structures has displayed good phototoxicity in EJ bladder carcinoma cells, with no dark toxicity.^[26]^ Nanoparticles built by self‐assembly of different‐sized hyperbranched polyethylene glycol (PEG) dendrimers covalently linked to the well‐known porphyrinoid pyropheophorbide‐a (Phe‐a) have shown a prolonged blood circulation time and deep tumor penetration and retention, which can be optimized by controlling the hydrophilic‐lipophilic balance of the macromolecular amphiphiles.^[27]^ Also, stable spherical nanoparticles of about 100 nm obtained by self‐assembly in aqueous solution of amphiphilic glycyrrhetinic acid‐modified Por have proved to be endocytosed by various cancer cells showing excellent PDT effect.^[28]^

**Figure 1:**
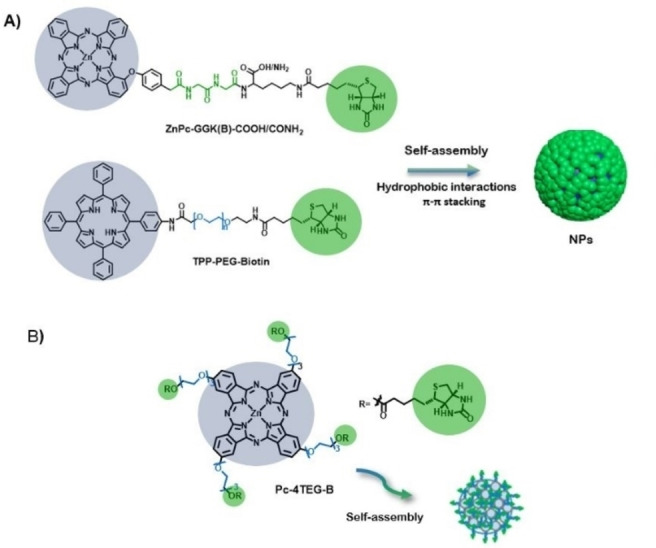
Representation of the chemical structure and aggregated nanoparticles (NPs) of A) **TPP‐PEG‐Biotin** ^[29]^ and **ZnPc‐GGK(B)‐COOH/CONH_2_**,^[31]^ and B) **Pc‐4TEG‐B**.^[30]^

Although passive targeting has undeniable advantages, including targeting labels in the PS structure increases the selectivity and affinity for specific biological targets of the final self‐assembled nanostructures, making them ideal delivery vehicles. Biotin and folic acid receptors are useful biomarkers for tumor‐targeting therapy, since they are overexpressed in many kinds of cancer cells ‐ such as brain, nose, ovary, breast, lung, colon, and prostate cancer‐ but scarcely expressed in normal cells. Pc and Por PS bearing targeting biotin groups have been prepared following an ”one‐for‐all” design. To impart amphiphilic features to the PS for the formation of nanoparticles in aqueous solution, either PEG[^29^, ^30^] or short peptide linkers^[31]^ are introduced between the PS and the biotin (Figure 1). Importantly, PEG not only affords hydrophilicity, but also minimizes the interactions of the nanoparticles with proteins or other biomolecules in the blood serum. Despite the different structure and, therefore, amphiphilic nature of the PS in Figure 1 (i. e., different substitution pattern and hydrophilic substituents), all the conjugates form uniformly sized, spherical nanoparticles in the 100–150 nm range. Biotin‐conjugated nanoparticles were tested in breast cancer cell lines MCF‐7,^[29]^ human hepatocellular carcinoma HepG2 cells and lung A549 cells mice tumors,^[31]^ ‐ all of them overexpressing biotin receptors‐ with excellent results.

**Figure 2:**
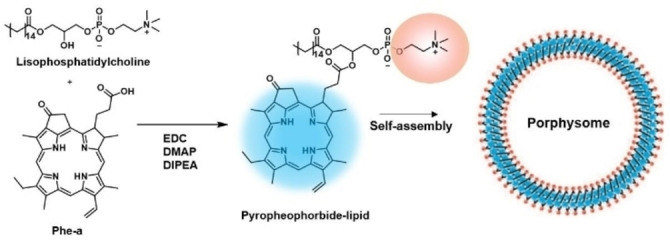
A) Synthesis of porphysome subunit, pyropheophorbide‐lipid (left) and the schematic representation of the assembled nanovesicle (right).^[33]^

A relevant approach developed by G. Zheng and co‐workers is the so‐called “ligand‐targeted porphysome design”.^[32]^ The term porphysome was used to denote the bilayered nanovesicles formed from self‐assembled porphyrin‐phospholipid conjugates.^[33]^ Porphysomes are outstanding examples of structurally well‐defined “one‐for‐all” nanosystems. The constituting porphyrin‐lipid conjugates were prepared by an acylation reaction between lysophosphatidylcholine and Phe‐a (Figure 2). Interestingly, the packing density of PS is very high (>80 000/particle), resulting in a very large nanostructure‐derived absorption, as well as a “super quenched” photoactivity (fluorescence and ^1^O_2_ production). However, folate receptor‐targeting porphysome constructed by including 1 mol% of folate‐PEG 2000‐lipid allows to turn these nanostructures to photoactive species due to porphysome disruption. Therefore, by folate‐receptor (FR) mediated endocytosis, folate‐porphysomes are internalized into cells, resulting in efficient disassembly of the nanostructures. After systematically administration to FR‐positive tumor‐bearing mice, folate‐porphysomes were capable to reduce the tumor burden by a PDT effect.

## 3 Responsive Nanomaterials

A step further to achieve selective therapeutic effects in the tumor tissues is to endow the self‐assembled nanoparticles with responsiveness to certain stimuli. Nanoparticles can be designed to be inactive during blood circulation and under normal physiological conditions, but to be activated inside the tumor interstitial space by acidic pH, enzymatic up‐regulation, light, temperature, redox environments or hypoxia once they extravasate into the tumor microenvironment. Indeed, stimuli‐triggered activation is highly desirable to minimize side effects to normal cells.^[34]^

Increased levels of ROS have generally been found in cancers, being H_2_O_2_ the most abundant species both in tumor cells and tumor microenvironment. This overproduction represents a useful tool for designing nanoassemblies for activatable phototherapy. For instance, a nanoprobe for luminescence imaging of tumors with highly expressed H_2_O_2_ has been assembled from an amphiphilic conjugate (defined as CLP) based on Chl e6 conjugated to luminol and PEG.^[35]^ Triggered by relevant levels of H_2_O_2_, CLP nanoparticles produced luminescence due to the luminol unit, and simultaneous excitation of Chl e6 by chemiluminescence resonance energy transfer, enabling *in vitro* and *in vivo* imaging of tumors and ^1^O_2_ generation for in situ PDT.

**Figure 3:**
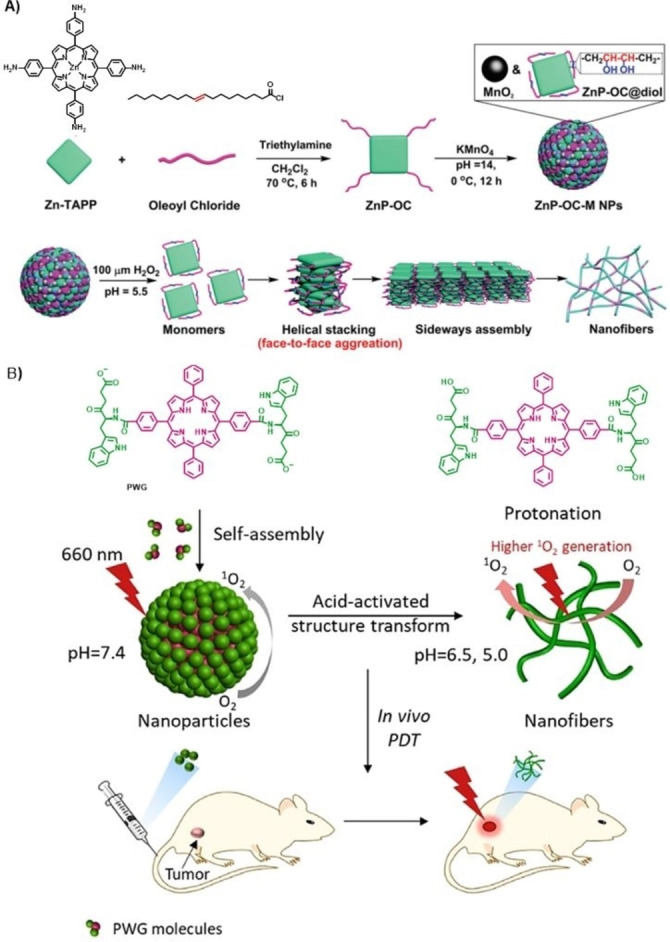
A) Preparation of **ZnP‐OC‐M NPs** (above) and schematic illustration for the supramolecular self‐assembled nanofiber formation (below).^[36]^ Adapted with permission from Ref. [36]. Copyright 2020, Royal Society of Chemistry. B) Schematic illustration of self‐assembly and fibrillar transformation of the acid‐activated peptide‐porphyrin (**PWG**) nanoparticles and their application in PDT.^[39]^ Adapted with permission from Ref. [39]. Copyright 2020, Wiley‐VCH.

Most tumor microenvironments are typically more acidic (pH around 6.5) than blood and extracellular fluid in healthy tissues. Moreover, the acidity is further increased in some subcellular compartments such as lysosomes (pH 4.5–5.0), which makes pH‐responsive nanostructures a useful design option for PDT. On the other hand, one important drawback for cancer treatments is the high tissue hydraulic pressure in tumor parenchyma that tends to pump the released drugs back to the systemic circulation. Z. Yuan and coworkers have designed a smart nanosystem to keep high the PS concentration in the tumor by improving its retention abilities.^[36]^ This strategy relies on the in‐situ self‐assembly capabilities of certain molecules through a response to the tumors environment that gives rise to an assembly‐induced retention effect, enhancing their biological function.^[37]^ The pH/H_2_O_2_‐responsive nanosystem (Figure 3A) consists in a Zn(II)‐meso‐tetraphenyl Por covalently linked to four oleic acid residues through amide bonds (ZnP‐OC), which is further modified by reaction with KMnO_4_ under alkaline conditions rendering the introduction of hydrophilic hydroxyl groups through addition reaction to the double bonds in the oleic chain. The resulting chiral ZnP‐OC@diol can self‐assemble in the presence of MnO_2_ to give *ca*. 100 nm sized ZnP‐OC‐M nanoparticles that exhibit self‐quenched fluorescence. After incubating HepG2 cells with ZnP‐OC‐M nanoparticles and, because of the pH/H_2_O_2_‐responsiveness of MnO_2_, disassembly occurs in the intracellular environment (pH=5.5, 100 μM H_2_O_2_). More importantly, the amphiphilic ZnP‐OC@diol molecules spontaneously assemble into nanofibers that exhibit excellent fluorescence emission and assembly‐induced retention effect. Meanwhile, the retention effect effectively increases the concentration of PS and enhances the PDT effect, suppressing the tumor development.

Peptides are interesting fragments to be linked to the PS because they afford responsiveness to pH, redox reactions, and enzymes. In this regard, peptide‐PS conjugates are candidates to self‐assemble into stimuli‐responsive nanomaterials displaying high anti‐tumor efficiency.^[38]^ In particular, a pH responsive peptide‐porphyrin conjugate, prepared by coupling the dipeptide tryptophan‐glycine (WG) to a hydrophobic Por (P) core via amidation, has proved its ability to form spherical self‐assembled nanoparticles under physiological conditions (Figure 3B).^[39]^ When the nanoparticles reach the tumor tissue, the protonation of PWG facilitates the formation of intermolecular hydrogen bonds, inducing the transformation of spheres into nanofibers, which show increased photoactivity (i. e., ^1^O_2_ generation) regarding the initial nanoparticles, as well as long‐term retention at tumor sites. Therefore, this is another example of transformable nanosystem that arranges itself in different assemblies because of an interplay between van der Waals interactions, hydrophobic effects, π‐π stacking interactions and hydrogen‐bonding, which are affected at different pH values.

An additional example of pH‐sensitive therapeutic nanoparticle, which behaves also as oxygen self‐supplying system has been developed by the self‐assembly of a tetrakis(pentafluorophenyl) Chl‐conjugated block copolymer.^[40]^ These nanoparticles have shown abilities to transport oxygen to a tumor, the oxygen loading realized by interactions with the fluorinated PS itself. The phototoxicity of the nanoparticles is greatly improved in an acidic aqueous environment due to the protonation of the amino groups present in the block copolymer, which weakens the aggregation of the PS.

Another type of stimuli that can activate the photophysical response of the PS is the endogenous glutathione (GSH) level. The concentration of GSH in the intracellular matrix is 100‐ to 1000‐fold higher than in extracellular fluids, and the GSH reducing reaction is a common redox system in cancer cells. Self‐assembled nanoparticles endowed with disulphide cross‐linkages may suffer disulfide reduction, triggering disassembly into theranostic – fluorescent and PDT active – PS, as explained in detail below.[^41^, ^42^] A remarkable example was reported by W. Zhang and coworkers, who reported Pors functionalized with dendritic branches through a disulfide linkage able to self‐assemble into spherical micelles in water.^[43]^ The uptake of these micelles by cells and release of the reduction‐sensitive PS in the cell interior was demonstrated.

Also, the presence of certain proteins such as albumin can induce disassembly of nanoparticles, leading to switchable photoactivity. Overexpressed albumin receptors on cancer cells have been suggested to facilitate the accumulation and degradation of albumin in tumor tissues.^[44]^ As a proof of concept, mono‐α‐4‐sulfonatophenoxyl Zn(II)Pc has been reported to self‐assemble in aqueous solutions to form regular vesicles with a diameter of approximately 15 nm, which display albumin‐dependent disassembly.^[45]^

## 4 Nanoassemblies for Photothermal Therapy

Cancer photothermal therapy (PTT) relies on the conversion of light energy (i. e. laser irradiation) into thermal energy to kill tumors and, as for PDT, it constitutes a spatio‐temporal controlled therapy.^[46]^ NIR‐absorbing nanoparticles that efficiently convert light energy into heat are commonly used for PTT. In addition to killing cancer cells, the photothermal effect can generate acoustic waves that can be detected and converted into imaging signals by an ultrasonic transducer, which is called photoacoustic imaging (PAI).^[47]^ This emerging diagnostic technique provides deeper penetration in biological tissue and higher resolution than traditional optical imaging techniques, and has been widely used for nanotheranostic purposes, as shown below. So far, a variety of photoactive nanomaterials, such as gold^[48]^ or polymeric‐based^[49]^ nanoparticles, among others, have been developed as photothermal agents (PTA). However, “one‐for‐all” photothermal nanoparticles based on porphyrinoid PS may overcome the limitations concerning fabrication processes, and potential biosafety of inorganic or polymeric PTAs.

Photothermal nanodots with an average value of 25 nm have been prepared from peptide‐containing Pors, showing high stability in aqueous media and under irradiation, as well as highly efficient light‐to‐heat energy conversion of 54.2 %.^[50]^ The peptide moieties in the conjugate provide aqueous stability to the nanodots, through hydrophilic interactions, but also a spatial barrier between Por groups to inhibit the further growth of the nanodots. The strong π‐stacking interactions in the assemblies produce the complete quenching of fluorescence and inhibition of generating ROS. Extensive in vivo evaluations demonstrate high biocompatibility and efficient accumulation and inhibition of the tumors for these nanodots through a PTT effect.

Porphysomes described by G. Zheng (Figure 2)^[33]^ were formerly used for PTT assays. As the packing density induced highly self‐quenched Por excited states, the absorbed energy is released as heat, making porphysomes exceptional as PTT agents.^[51]^ Porphysomes and Photofrin were injected intravenously into mice bearing dual xenograft tumors to directly compare PDT and PTT. They demonstrated the nanostructure‐driven conversion from a PDT mechanism of the PS alone to a completely thermal mechanism of the porphysomes. Using a hypoxia tumor model, these nanostructures were found advantageous to overcome hypoxic conditions and achieve effective ablation of solid tumors.

**Figure 4:**
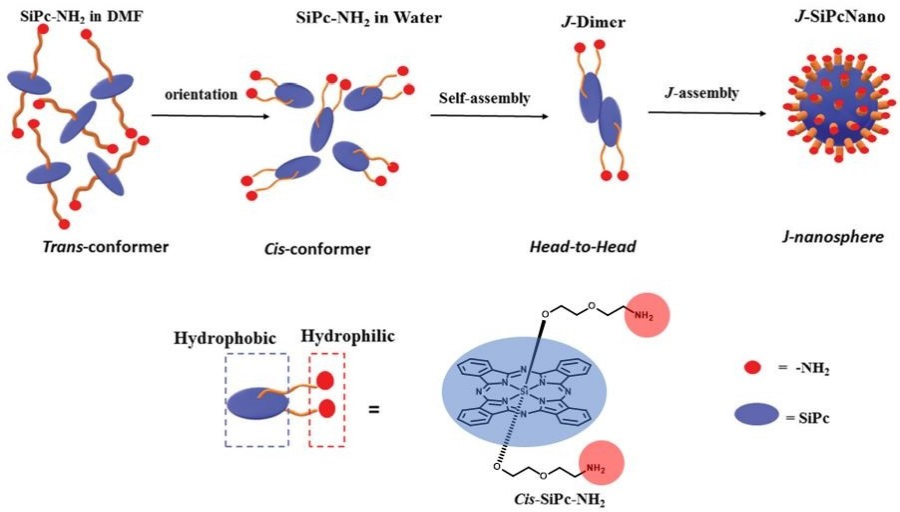
Schematic representation of the self‐assembly mechanism of J‐SiPcNano formation in water.^[53]^ Adapted with permission from Ref. [53]. Copyright 2020, Royal Society of Chemistry.

PTT can operate in combination with PDT (and other cancer therapies) to achieve synergistically enhanced therapeutic efficacy. The synergistic effect of PDT and PTT has been found in structurally simple models, such as tetra (4‐carboxyphenyl)porphyrin, which self‐assembles into water‐soluble sphere–like nanoparticles with high photothermal conversion efficiency (η=31 %) and long photothermal stability.^[52]^ The synergistic PDT/PTT effect was proved *in vitro* in HeLa cells either by mixing vitamin C (regarded as inhibitor for PDT through reduction of ROS) with the nanoparticle solution before incubation, or by using an ice bath to control PTT. In both cases, the cells showed high survival rate. Another example of phototherapeutic nanomedicine with combined PDT and PTT properties has been described by J. Jiang and coworkers, who prepared a Si(IV)Pc with two axial NH_2_‐terminated diethylene glycol ligands able to self‐assemble through their cis‐conformation in *J*‐type aggregates in the form of monodisperse regular nanospheres with a diameter of *ca*. 100 nm (Figure 4).^[53]^ This type of aggregation results in a red‐shifted absorption (750–850 nm) and unquenched fluorescence emission, as well as photodynamic activity. Both *in vitro* and *in vivo* therapeutic results unveil the effective near‐infrared PDT/PTT synergistic effect for cancer treatment and fluorescence imaging (FI) of SiPc‐based nanoparticles. Nanoparticles obtained from ZnPcs tetra‐functionalized in their alfa‐position with 6‐hydroxylhexyloxy chains also exhibited high photothermal conversion efficiency (as high as 31.3 %) and remarkable PDT performance.^[54]^

## 5 Nanotheranostic Systems

The term theranostics embodies the concepts of therapeutics and diagnostics. PDT can be inherently considered as a theranostic modality as the molecules involved are photochemically active, leading to the desired therapeutic effect (^1^O_2_ generation), and in many cases, to a fluorescent emission in an appropriate wavelength that enables the diagnosis component. The main advantage of theranostic photomedicines is that permits to realize personalized treatments in different ways, namely, individualizing PDT dosimetry by monitoring PS fluorescence, using the PS fluorescence properties to guide surgery, or monitoring the treatment efficiency, which may include other imaging modalities such as magnetic resonance imaging (MRI) or PAI. Nevertheless, attaining high performance of these functions *in vivo* in one single nanoconstruct is extremely challenging.

With a focus on imaging‐guided tumor PDT applications, star‐like, hydroxyl‐rich polycations with flanking Pcs have been examined as candidates for PAI‐guided antitumor therapy.^[55]^ The designed polymers were prepared from a ZnPc core tetra‐functionalized with ethanolamine units. The four terminal amino groups were converted into atom‐transfer radical polymerization (ATRP) initiation sites, which allow to further carry out the polymerization with glycidyl methacrylate. Then, poly(glycidyl methacrylate) arms were further functionalized with amino‐containing ZnPcs. The strong π‐π stacking and hydrophobicity of the Pc units drive the amphipathic polymers to self‐assemble into well‐defined cationic nanoparticles. Such Pc‐PGEA/Pc nanoparticles present impressive PDT effects under moderate irradiation and remarkable PAI ability.

**Figure 5:**
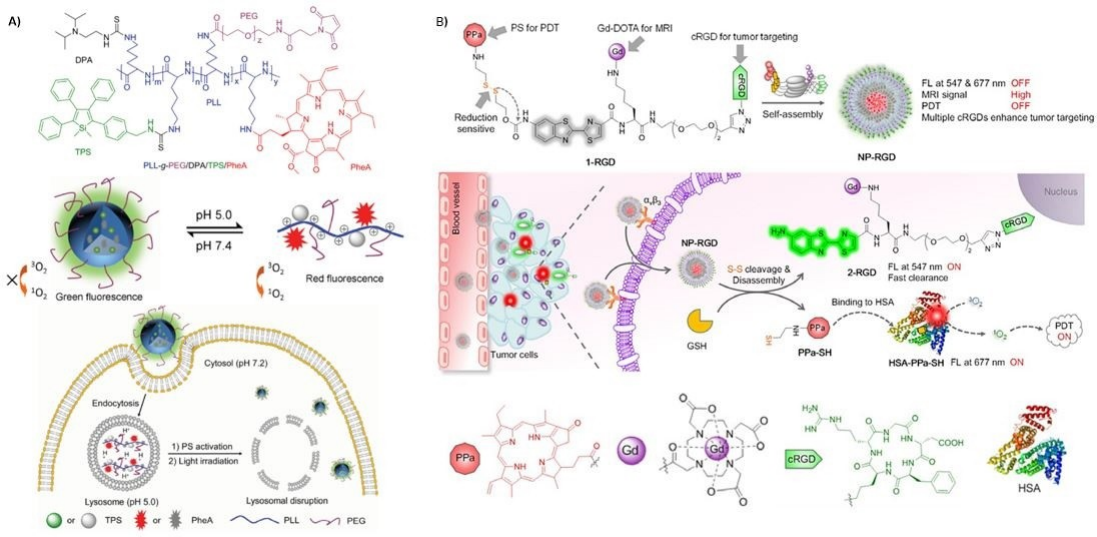
A) Chemical structure of **PLL‐g‐PEG/DAP/TPS/PheA** (above); Schematic illustration of the pH‐activation and its use for self‐tracking, cancer cell imaging, phototoxicity restoration in the acidic lysosome, and in situ monitoring of lysosomal membrane disruption as an indicator of therapeutic response and cell death prediction (below).^[56]^ Adapted with permission from Ref. [56]. Copyright 2015, Wiley‐VCH. B) Proposed self‐assembled structure of **NP‐RGD** (above); Schematic illustration of **NP‐RGD** for on demand PDT via activation by intracellular GSH and albumin (middle); Chemical structures of the PPa, DOTA‐Gd chelate, cRGD and a cartoon illustration of HSA structure (below).^[41]^ Adapted with permission from Ref. [41]. Copyright 2020, Wiley‐VCH.

As mentioned above, lysosome is an important cellular organelle featuring weak acidic environment (pH 4.5–5.0), which has been utilized as a target for cancer diagnosis and pH‐responsive drug delivery. An interesting pH‐responsive nanoprobe for tracking the whole process of cancer therapy takes advantage of the opposite photophysical properties of aggregation‐induced emission fluorogens (AEIgens), and PS exhibiting aggregation‐caused quenching (ACQ) features (Figure 5A).^[56]^ This molecular probe (PLL‐g‐PEG/DAP/TPS/PheA) comprises poly‐L‐lysine grafted with a diisopropylamino side group, a tetraphenylsilole AIEgen, an ACQ Phe‐a PS, and a PEG‐maleimide block for further conjugation to a targeting ligand, namely, cyclic arginyl‐glycyl‐aspartic acid peptide sequence (cRGD), which can specifically recognize the cell surface receptor integrin α_v_β_3_. The self‐assembled probe forms spherical nanoparticles of *ca*. 100 nm, exhibiting low phototoxicity and green fluorescence (AIEgen emission) at physiological conditions (pH 7.4), where the Phe‐a molecules are aggregated, and the red fluorescence is quenched. The nanoprobe was incubated in MDA‐MB‐231 cells with overexpressed α_v_β_3_ integrin and entrapped in the acidic lysosomes. Due to protonation of diisopropylamino groups the nanoprobe is disassembled and, as a result, the green fluorescence is weakened, while the red fluorescence as well as phototoxicity from Phe‐a is restored, which can be used for monitoring PS activation. Upon light irradiation, Phe‐a induces ROS generation which causes lysosomal membrane disruption and leakage of the probe to the cytoplasm (pH 7.2). Consequently, the probe assembles again and emits green fluorescence from the tetraphenylsilole AIEgen, thus reflecting the lysosomal membrane disruption as an indication of the therapeutic response.

Fluorescence‐,^[57]^ PA‐,^[58]^ and MRI‐guided^[59]^ PTT has been also explored. Interestingly, seminal combination of contrast and PTT agents into a single nanostructure was described in porphysomes in which the Phe‐a unit chelates paramagnetic Mn^3+^ions.^[59]^ The fact that all the constituting Phe‐a units in the porphysome (around 80.000) are labelled gives rise to a very potent contrast agent. Interestingly, metallation of the macrocycle synergistically improves the already potent PTT capabilities of free‐base porphysomes.

A sophisticated redox‐responsive, magnetic and fluorogenic porphyrinoid nanoconstruct (NP‐RGD) has been recently described via self‐assembly of a disulfide‐containing amphiphilic molecule comprising a Phe‐a PS for FI and PDT, a disulfide bond as a redox‐cleavable linker, a pre‐quenched amino oxyluciferin fluorophore, a paramagnetic gadolinium‐tetraazacyclododecanetetraacetic acid (DOTA‐Gd) chelate for MRI; and a cRGD sequence for targeted therapy (Figure 5B).^[41]^ This smart molecular probe self‐assembles in aqueous solution into monodisperse 60 nm sized nanoparticles, which show quenched fluorescence and PDT activity. Given the large molecular size of the Gd‐containing nanoparticles, their r_1_ relaxivity is higher than that of commercial contrast agents, producing a bright T1‐weigthed MR image.

**Figure 6:**
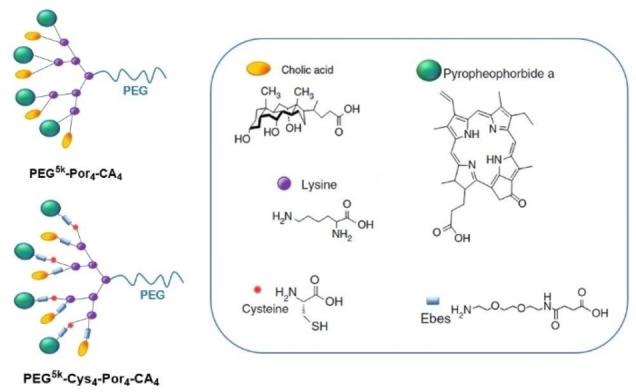
Schematic illustration of **PEG^5k^‐Por_4_‐CA_4_**, the cross‐linkable porphyrin‐telodendrimer **PEG^5k^‐Cys_4_‐Por_4_‐CA_4_**, and the chemical structure of the building blocks that conform the telodendrimers.^[60]^

Another innovative and versatile “one‐for‐all” Por‐based nanomedicine was constructed by using an amphiphilic polymer (called telodendrimer PEG^5k^‐Por_4_‐CA_4_) comprising a linear PEG and dendritic oligomers of the porphyrinoid Phe‐a and cholic acid (CA) (Figure 6).^[60]^ This hybrid polymer self‐assembles in 20 nm nanoparticles in PBS, with self‐quenched emission and photodynamic transduction, which can be both restored upon the addition of SDS that triggers the disassembly. On the other hand, the nanoparticles also yield efficient photothermal transduction upon illumination. A more sophisticated version of these nanoparticles includes a reversible disulphide crosslinking strategy. Introducing four cysteines to the oligolysine backbone of the telodendrimer, to form PEG^5k^‐Cys_4_‐Por_4_‐CA_4_, results in nanoparticles that can be further crosslinked via disulphide bond through oxidation of the thiol groups, yielding more stable 32 nm‐sized spherical vesicles that disassemble under incubation with GSH. This type of nanoassemblies show ability to chelate imaging agents such as ^64^Cu(II) and Gd(III), and to encapsulate anticancer drugs such as DOX, that can be then released upon addition of GSH. Experiments in both ovarian cancer xenograft model and murine transgenic breast cancer model demonstrated that these novel nanoparticles could be used as both nanoprobes with increased sensitivity of multimodal imaging for tumor detection and nanotransducers, which can be activated to generate heat and ROS efficiently at sites for PTT/PDT dual therapy via a single‐wavelength light. The tumor superior imaging capability of the nanoparticles can be further utilized to monitor their real‐time *in vivo* delivery and for assessing their therapeutic efficacy.

**Figure 7:**
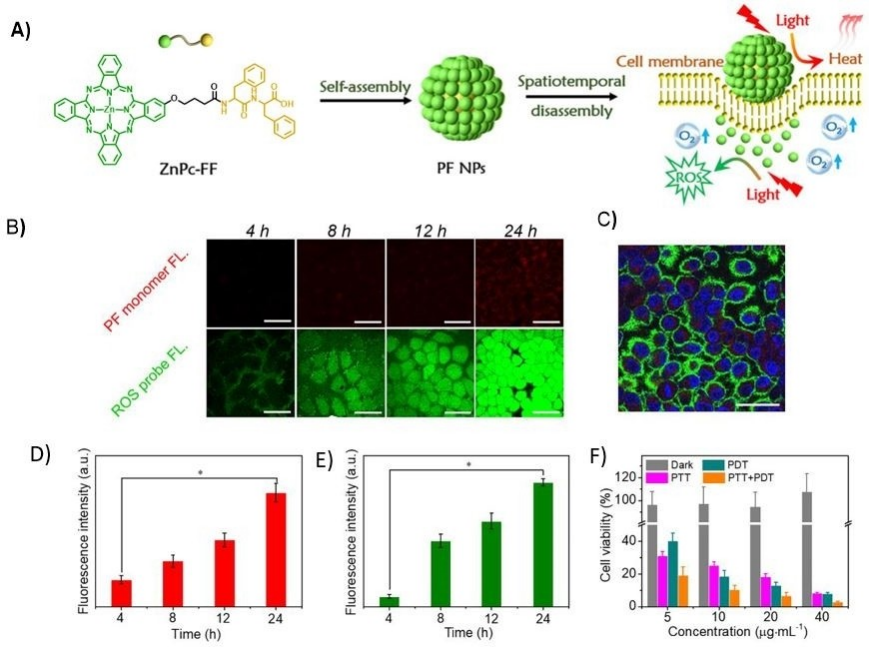
A) Schematic illustration of the spatio‐temporally coupled photoactivity of **PF** self‐assemblies for localized adaptive tumor theranostics. B) CLSM images of MCF‐7cells incubated with **PF** nanoparticles (**PF NPs**). Red fluorescence was from monomeric Pc molecules. Green fluorescence showed the generated ROS *in situ* within cells using DCFH‐DA as an indicator. C) image of MCF‐7 cells incubated with **PF NPs** for 24 h D) Fluorescence intensity obtained from the monomeric Pc molecules within MCF‐7 cells in Figure A). E) Fluorescence intensity obtained from the ROS probe within MCF‐7 cells in Figure A). F) Viability of MCF‐7 cells incubated with **PF NPs** in the dark and under laser irradiation.^[61]^ Adapted with permission from Ref. [61]. Copyright 2019, Wiley‐VCH.

An interesting combination of PAI/PTT and FI/PDT theranostic modalities have been found for Pc‐(L‐phenylalanine‐L‐phenylalanine) (PF) one‐component nanoparticles, which can be integrated in a spatio‐temporally coupled manner (Figure 7).^[61]^ Strong hydrophobic interactions combined with π‐π stacking promote PF self‐assembly into well‐defined nanoparticles with excellent colloidal stability along with supramolecular PTT effects, but with completely quenched fluorescence emission. Interestingly, upon incubation of MCF‐7 cells with PF nanoparticles, cell membrane‐triggered disassembly behavior was observed by Pc‐red fluorescence signal emergence. The disassembled Pc monomers were localized in the cytoplasm, and intracellular production of ROS was probed. To prove adaptive photo‐theranostic behavior of the PF nanoparticles, they were injected into MCF‐7 solid tumors. PA signal remained strong for 4 h and, from then on, the fluorescence gradually increased, with the simultaneous decrease of the PA signal, until complete dispersion in the tumor site at 24 h. Concomitantly, PTT effect is dominant within 4 h, while PDT can be conducted at 24 h, providing a switchable, effective strategy for localized tumor phototherapy. PAI/PTT and FI/PDT theranostic modalities have been also proved with nanostructured ZnPc assemblies in biotin‐receptor positive A549 cells and tumors.^[25]^ Fluorescence and ROS generation can be triggered because of the biotin‐receptor‐induced, partial disassembly of the ZnPc nanoparticle, which creates opportunities for low‐background FI and activatable PDT, although PTT and PAI abilities remain due to certain survival of the assemblies.

## 6 Nanoassemblies for Combined Chemo/Photodynamic Therapy

Multidrug resistance (MDR), whether inherent or acquired, greatly threatens chemotherapy clinical outcomes. Also, chemo‐drugs are not able to distinguish normal cells from cancer cells, resulting in serious side effects due to the lack of selectivity. Combination therapy (chemo‐photodynamic) has been suggested as an alternative strategy to reverse MDR and minimize side effects through different mechanisms of action.

**Figure 8:**
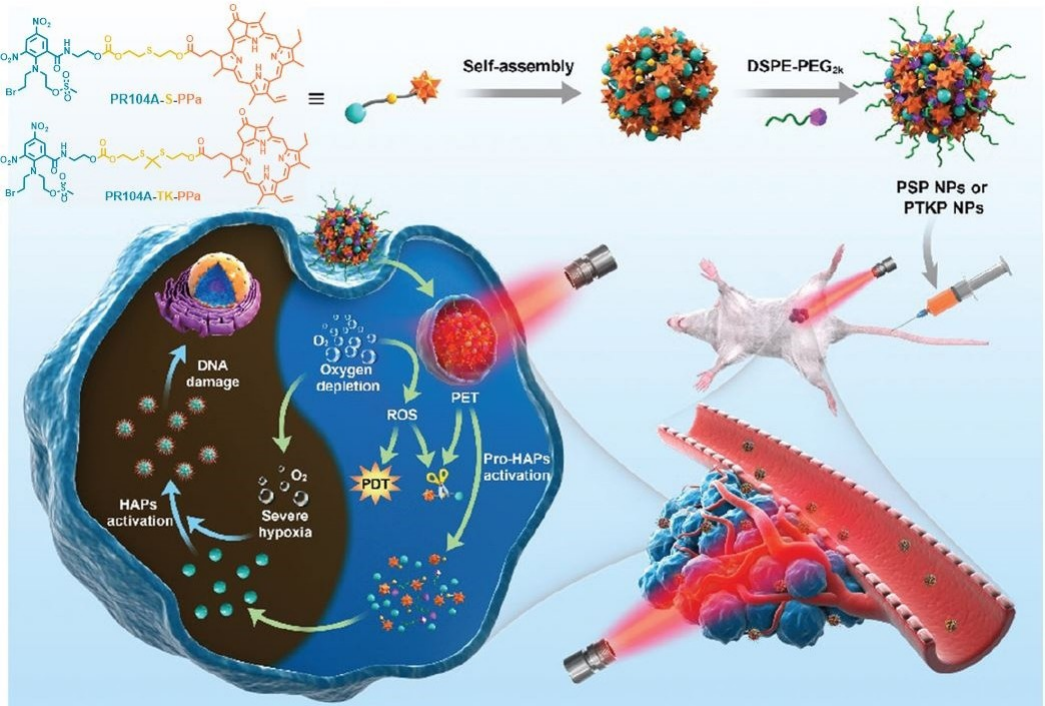
Schematic representation of the formation of heterodimeric prodrug‐nanoparticles (PSP NPs and PTKP NPs), their light‐triggered dual‐modality activation, and the PDT‐induced activation of the HAP **PR104A**.^[62]^ Adapted with permission from Ref. [62]. Copyright 2020, Royal Society of Chemistry.

Light‐triggered drug release of chemotherapeutic drugs from nanodelivery systems allows for a precise temporal and spatial feeding to the tumor cells. Smart heterodimeric molecules reported by Z. He and J. Sun are formed by combination of hypoxia‐activated prodrug (HAP) PR104A to the Phe‐a porphyrinoid coded as PPa through a thioether (PR104A‐S‐PPa) or thioketal (PR104A‐TK‐PPa) linkage, which self‐assemble into uniform nanoparticles (Figure 8).^[62]^ Interestingly, light‐induced disassembly takes place by photoinduced electron transfer (PET) from PPa to the neighboring sulfur atoms of the bridges to form sulfur radical cations, which eventually yield the cleavage of C−S bonds, the release of PR104A, and the subsequent disintegration of prodrug‐nanoparticles. Then, ROS can be generated through the reaction between excited PPa and surrounding O_2_, activating PDT therapeutic effects.

**Figure 9:**
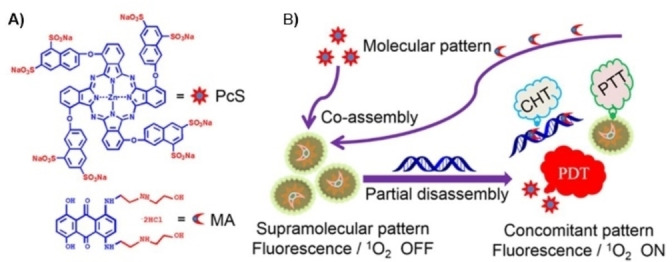
A) chemical structure of octasulfonated phthalocyanine (**PcS**) and mitoxantrone (**MA**) B) Schematic illustration of the co‐assembly between **PcS** and **MA** to form a nanotheranostic agent and its nucleic‐acid‐driven activatable properties for fluorescent imaging and PDT synergized with PTT and CHT.^[63]^ Adapted with permission from Ref. [63]. Copyright 2018, American Chemical Society.

Another stimuli‐responsive supramolecular nanostructure, comprising a PS and an anticancer agent has been reported by J. Yoon and coworkers,^[63]^ which consist in the co‐assembly of 6,8‐disulfonate‐2‐naphthyloxy ZnPcs (PcS) with the anticancer drug mitoxantrone (MA) (Figure 9). Mixing MA and PcS in water yields nanoassemblies with a mean hydrodynamic diameter of about 60 nm that prove stable over long periods of time. The nanoparticles show cancelled emission and ^1^O_2_ generation abilities in water but work as PTAs converting light into heat energy. The authors observed that disassembly takes place upon mixing the nanostructures with calf thymus DNA, triggered by the large binding constant between both, with a concomitant fluorescence and ^1^O_2_ generation upturn, but keeping a mild photothermal behavior. Encouraged by the DNA‐responsive properties of the material, the authors explored the activatable photoactivities of PcS‐MA in MCF7 cancer cells and tumor‐bearing mice. *In vivo* evaluations indicate that PcS‐MA nanostructures have a high level of accumulation in tumor tissues. Interestingly, PcS‐MA show cytotoxic effect under laser irradiation through a synergic PDT, PTT and chemotherapeutic effect (CHT). The improved chemotherapeutic effect was demonstrated by mice treatment with PcS‐MA without laser irradiation. The found tumor growth inhibition (44.7 %) was better than that produced by treatment with MA alone (22.8 %), indicating that this approach strengthens the chemotherapeutic effect of MA because of its increased accumulation and, therefore, prolonged action in tumors. Tirapazamine, which is used as a hypoxia‐activated chemotherapy prodrug, has been also co‐assembled with a PEGylated Chl e6.^[64]^

Particularly interesting is the use of self‐assembled nanophotosensitizing systems implemented with target‐specific residues and showing high loading efficiency for the encapsulation of chemotherapeutic drugs. These nanoconjugates may exhibit high cellular uptake and enhanced tumor accumulation, and synergistic anticancer effect with PDT action. In this regard, ZnPc molecules conjugated with short peptide (Figure 1B)^[31]^ or Por conjugated with PEG chains (Figure 1A),^[24]^ both capped with a biotin termini, have shown to encapsulate DOX during the self‐assembly. The resulting co‐assembled nanoparticles have shown preferential uptake toward the biotin‐receptor‐positive HepG2 cells,^[31]^ and breast cancer cell line MCF‐7.^[29]^ Disassembly of the nanoparticles upon internalization leads to DOX release, thereby affording synergistic dual chemo‐ and photodynamic therapy. Other nanocarriers able to encapsulate DOX, with programmable releasing ability triggered by GSH present at the tumor site and with nanotheranostic capabilities have been described above.^[60]^

## 7 Nanoassemblies with Antimicrobial Activity

Within the wide field of phototherapy, a lot of attention has been focused on the application of PDT for the treatment of infectious diseases.^[65]^ Nowadays, the therapy of infectious diseases is increasingly challenged by the large number of pathogens combated, as well as by the continuous inception of multi‐drug‐resistant pathogens. Antimicrobial PDT (a‐PDT) is particularly suitable for the treatment of localized microbial infections, including antibiotic‐resistant microbial strains. Gram‐positive bacteria have shown to be highly susceptible to a‐PDT, while the inactivation of gram‐negative bacteria has proved more challenging due to the impermeability properties of their outer membrane. Cationic porphyrinoids are usually more efficient than neutral or anionic dyes,^[66]^ which is particularly true for gram‐negative bacteria, due to the presence of a greater number of negative surface charges that attract cationic PS, facilitating the diffusion process. In this regard, cationic nanoparticles strongly interact with the cell membranes, thereby enhancing their intracellular uptake.^[67]^

**Figure 10:**
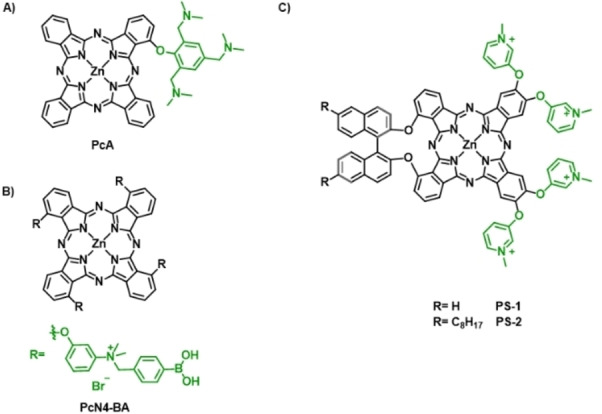
The chemical structure of A) **PcA**,^[68]^ B) **PcN_4_‐BA**,^[69]^ and C) **PS‐1** and **PS‐2**.^[73]^

Positively charged ZnPcs have proved particularly effective in killing both gram‐positive and gram‐negative bacteria, particularly when they self‐assemble in cationic nanoparticles. Introduction of amine groups susceptible to protonation or quaternization is beneficial to enhance hydrophilicity of PS, providing them also with the ability to self‐assemble in nanostructures with a positively charged surface to target negatively charged pathogen cell membranes. J. Yoon and coworkers have selected 2,4,6‐tris‐(*N*,*N*‐dimethylaminomethyl)phenoxy substituted ZnPc (PcA) as building block for self‐assembly (Figure 10A).^[68]^ Both Transmission Electron Microscopy (TEM) and Dynamic Light Scattering (DLS) studies reveal that PcA forms stable spherical nanoparticles in aqueous media of about 50 nm and zeta potential of +30 mV. Notably, the nanoparticles show quenched fluorescence and do not display photothermal effects but promote ROS generation through a type‐I mechanism. This novel nanoPS displays excellent a‐PDT ability against *E. coli* and *S. aureus*, as respective representatives of gram‐negative and gram‐positive bacteria, along with their corresponding antibiotic‐resistant bacterial strains. Going one step further,^[69]^ the same authors report on the aggregation‐enhanced photodynamic effect of a 3‐{*N*‐(4‐boronobenzyl)‐*N,N*‐dimethylammonium} phenoxy‐substituted ZnPc (PcN4‐BA) (Figure 10B). The selection of boronic acids as end groups relies on previous findings that point out boronic acid as a useful targeting group for bacterial binding because of its ability to form a pair of covalent bonds with glycans on the surface.^[70]^ Interestingly, enhanced ROS generation through both type I and type II photochemical mechanisms was observed for PcN4‐BA aggregates with respect to the monomeric species, due to an enhanced intersystem crossing process. In addition, PcN4‐BA displays excellent a‐PDT activity against both common and antibiotic‐resistant bacterial strains. Also, co‐assemblies of tetrasulfonate ZnPcs with positively charged diazobenzenes (NanoAzoPcs) show a‐PDT that can be controlled by varying the stoichiometric ratios of the components and using isomerization of azobenzene.^[71]^

A novel archetype of amphiphilic Pc‐based PS relies on a binaphthyloxy‐linked A_2_B_2_ ZnPc core structure (with A and B coding for two differently functionalized isoindoles),^[72]^ functionalized with pyridinium‐substituents at two of the isoindole to provide water solubility.^[73]^ In particular, two PS endowed with different amphiphilic character were prepared, either attaching two octyl chains at the binaphthyl unit (PS‐2) or keeping the ring bare (PS‐1) (Figure 10C). These molecules showed ability to form positively charged and stable nanoparticles in aqueous solution, and to disassemble in more hydrophobic media. Both nanostructures show self‐quenched photoactivity in aqueous media, because of the formation of *H*‐type aggregates that deactivate the excited states of the chromophores. However, binding to the negative outer membrane of Gram‐positive and Gram‐negative bacteria triggered a disassembly of the nanoparticles, which produced an efficient inactivation after light irradiation. Particularly, *E. coli* assays showed an unusually high photoactivity of the PS‐1 in killing this type of resistant bacteria. Compared to other tetracationic pyridinium PS, the oriented amphiphilic character, and thus, the self‐assembly behavior of PS‐1 and PS‐2, provide them with much stronger a‐PDT activity both in terms of concentration of the PS and light dose.

## 8 Summary and Outlook

It is manifest that conventional PDT using free PS is not the most effective choice for cancer management. Unquestionably, nano‐delivery systems are essential to improve transport and tumor targeting efficacy. In addition, formulations that include the prospect of combined therapy and image‐guidance are valued to improve the clinical outcome. In this regard, the “one‐for‐all” concept is gaining ground in the extensive field of photonanomedicines, since it overcomes limitations concerning multistep fabrication and low reagent loadings of traditional delivery systems based on inorganic or polymeric nanoparticles. This approach relies on the rational design of PS combining self‐assembly capabilities for the straightforward preparation of stable, nanometer‐sized structures, with therapeutic functions and, preferably, imaging, and active targeting abilities. Multifunctionality has been successfully achieved in some of the reported examples, which describe smart nanostructures able to overcome *in vivo* biological barriers and to deliver the PS/imaging agent efficiently in a targeted manner to diseased tissues. The fact that single component nanoassemblies show such a high performance is extremely encouraging in the search of readily available and efficient photomedicines.

## Conflict of interest

The authors declare no conflict of interest.

## Biographical Information

*Irene Paramio received her Master's degree (2020) from Universidad Autónoma de Madrid (UAM). She is currently pursuing her PhD at UAM under the supervision of Dr. G. de la Torre and Prof. T. Torres. Her research interests focus on the preparation of phthalocyanine‐based self‐assembled multifunctional nanosystems for cancer phototherapy*.

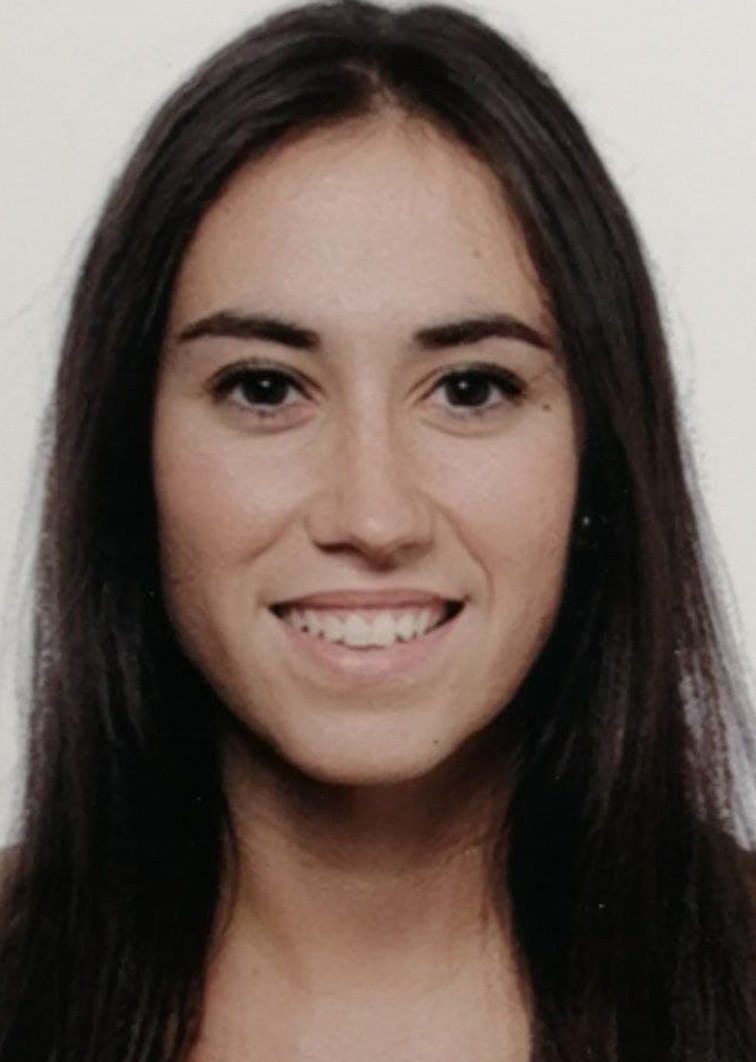

## Biographical Information

*Tomás Torres is Head of the Institute for Advanced Research in Chemical Sciences (IAdChem), Full Professor of Organic Chemistry at the Autónoma University of Madrid (UAM) and Associated Senior Scientist at IMDEA Nanoscience. In addition to various aspects of synthetic and supramolecular chemistry, his current research interests include the preparation and study of optical properties of organic materials based on phthalocyanines, and their applications in molecular photovoltaics, and photodynamic therapy, with a focus on nanotechnology*.

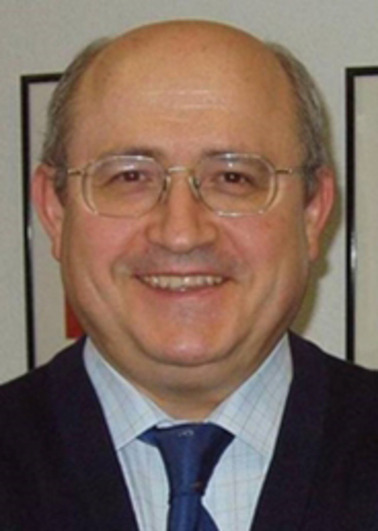

## Biographical Information

*Gema de la Torre graduated in Chemistry (1992) and obtained her PhD (1998) from Universidad Autónoma de Madrid (UAM). Currently she is Associate Professor in the Organic Chemistry Department at UAM. Her current research interests are the synthesis of photophysically active, porphyrinoid‐based metalloorganic complexes and nanostructures, with application in organic photovoltaic devices, catalysis, and photodynamic therapy*.